# The effect of antiretroviral therapy provision on all-cause, AIDS and non-AIDS mortality at the population level – a comparative analysis of data from four settings in Southern and East Africa

**DOI:** 10.1111/j.1365-3156.2012.03032.x

**Published:** 2012-07-30

**Authors:** Sian Floyd, Milly Marston, Kathy Baisley, Alison Wringe, Kobus Herbst, Menard Chihana, Ivan Kasamba, Till Bärnighausen, Mark Urassa, Neil French, Jim Todd, Basia Zaba

**Affiliations:** 1 London School of Hygiene and Tropical MedicineLondon, UK; 2 Africa CentreKwaZulu Natal, South Africa; 3 Karonga Prevention StudyKaronga, Malawi; 4 Medical Research CouncilEntebbe, Uganda; 5 Harvard School of Public HealthBoston, MA, USA; 6 National Institute of Medical ResearchMwanza, Tanzania

**Keywords:** antiretroviral therapy, mortality, sub-Saharan Africa

## Abstract

**Objective:**

To provide a broad and up-to-date picture of the effect of antiretroviral therapy (ART) provision on population-level mortality in Southern and East Africa.

**Methods:**

Data on all-cause, AIDS and non-AIDS mortality among 15–59 year olds were analysed from demographic surveillance sites (DSS) in Karonga (Malawi), Kisesa (Tanzania), Masaka (Uganda) and the Africa Centre (South Africa), using Poisson regression. Trends over time from up to 5 years prior to ART roll-out, to 4–6 years afterwards, are presented, overall and by age and sex. For Masaka and Kisesa, trends are analysed separately for HIV-negative and HIV-positive individuals. For Karonga and the Africa Centre, trends in AIDS and non-AIDS mortality are analysed using verbal autopsy data.

**Results:**

For all-cause mortality, overall rate ratios (RRs) comparing the period 2–6 years following ART roll-out with the pre-ART period were 0.58 (5.9 *vs*. 10.2 deaths per 1000 person-years) in Karonga, 0.79 (7.2 *vs*. 9.1 deaths per 1000 person-years) in Kisesa, 0.61 (6.7 compared with 11.0 deaths per 1000 person-years) in Masaka and 0.79 (14.8 compared with 18.6 deaths per 1000 person-years) in the Africa Centre DSS. The mortality decline was seen only in HIV-positive individuals/AIDS mortality, with no decline in HIV-negative individuals/non-AIDS mortality. Less difference was seen in Kisesa where ART uptake was lower.

**Conclusions:**

Falls in all-cause mortality are consistent with ART uptake. The largest falls occurred where ART provision has been decentralised or available locally, suggesting that this is important.

## Introduction

Adult HIV prevalence has been high across Southern and East Africa since the 1990s, ranging from around 6% to over 20% (UNAIDS & WHO 2009), and recent evidence shows that HIV incidence remains high (Stover *et al.* 2010). The impact of HIV on adult mortality has been huge: for example, 63% of all deaths were attributed to AIDS among 15–59 year olds in northern Malawi (Jahn *et al.* 2008) between 2002 and 2005, around half of all deaths in 15–44 year olds in a rural community in Mwanza, Tanzania were because of AIDS during 1994–1998 (Urassa *et al.* 2001) and 60% of all deaths were attributable to AIDS in KwaZulu Natal, South Africa between 2000 and 2003 (Hosegood *et al.* 2004).

Since about 2004, provision of antiretroviral therapy (ART) has been rolled out in the public sector across sub-Saharan Africa. ART has the potential to prolong and save lives on a massive scale. Median retention in care among individuals in ART programs in Africa after 3 years is around 70% (Fox & Rosen 2010), and there is accumulating evidence that this can translate into substantial reductions in adult mortality at the population level (Herbst *et al.* 2009; Reniers *et al.* 2009; Floyd *et al.* 2010; Mwagomba *et al.* 2010; Kasamba *et al.* 2012; Marston *et al.* 2012). For example, in Karonga district in northern Malawi, all-cause mortality among 15–59 year olds fell by around 30% and mortality attributed to AIDS by around 50% during the third year of ART provision, compared with the years immediately preceding ART roll-out (Floyd *et al.* 2010). In KwaZulu Natal, among adults aged 25–49 years old, mortality attributed to AIDS fell by around 25% averaged over the first 3 years of ART roll-out (Herbst *et al.* 2009), and in Masaka district, Uganda all-cause mortality among 15–59 year olds fell by an average of around 40% between 1 and 5 years after the start of free ART provision (Kasamba *et al.* 2012).

The population-level effect of ART provision on adult mortality will vary across settings, depending on HIV prevalence, access to ART and retention in care after starting treatment. In this paper, we synthesise evidence across four demographic surveillance sites (DSS), in Malawi, South Africa, Tanzania and Uganda. Our aim is to provide a broad and up-to-date picture of the effect of ART on population-level mortality in Southern and East Africa, using the same methods of analysis across sites to enable fair comparison. We analyse time trends in all-cause adult mortality, overall and also stratified according to an individual’s HIV status for two sites (Masaka and Kisesa) with high coverage of HIV testing in the DSS both before and following ART roll-out. We also analyse AIDS and non-AIDS mortality for two sites (Karonga and the Africa Centre), which have verbal autopsy data available for analysis for both the time period immediately preceding ART roll-out and subsequently. We interpret the findings in the context of ART uptake and retention in treatment programmes in each site.

## Methods

### Sites and settings

The Karonga DSS is located in rural northern Malawi, was established with a baseline census in 2002 and has a total population of around 33 500 (Jahn *et al.* 2007). Most of the population are subsistence farmers, and adult HIV prevalence is approximately 10% (McGrath *et al.* 2007). The Kisesa DSS and cohort study is located in north-west Tanzania, was established in 1994 and has a total population of around 30 000 (Mwaluko *et al.* 2003). Most of the population are subsistence farmers, but petty trading is also an important income source, and adult HIV prevalence is around 6% (Zaba *et al.* 2010). The Masaka DSS is located in rural south-west Uganda, was established in 1989, has a total population of around 20 000 (Mbulaiteye *et al.* 2002), and an adult HIV prevalence of 7–8% (Shafer *et al.* 2008). The Africa Centre DSS is in rural KwaZulu Natal, South Africa, and was established in 2000, when it had a total population of around 90 000 (Tanser *et al.* 2008). Adult HIV prevalence was around 22% among 15–54 year olds in 2004 (Welz *et al.* 2007).

### Demographic surveillance

In Karonga and Masaka DSS, information on births and deaths is collected monthly, and in- and out-migrations are updated during an annual re-census (Jahn *et al.* 2007; Kasamba *et al.* 2012). In Kisesa and the Africa Centre DSS, data on births, deaths and migrations are collected at 6-month intervals (Mwaluko *et al.* 2003; Tanser *et al.* 2008).

### HIV testing as part of the research programme, Kisesa and Masaka

In Kisesa, sero-surveys have been conducted approximately every 3 years, since 1994, with informed consent. Six sero-surveys have been completed, with participation rates of 63–74%, and all individuals who are resident in the DSS area and aged 15 years or more are eligible to participate. HIV testing is carried out at a temporary clinic established in the centre of each village in the DSS; since 2003, all individuals who wish to learn their HIV status have been referred to a counsellor who is a member of the sero-survey team.

In Masaka, annual sero-surveys among individuals aged 13 or more years old started in 1989, and are conducted at people’s homes. Individuals can obtain the result of the HIV test several weeks later, from a local VCT centre. Around 60–65% of all eligible individuals consent to HIV testing in each sero-survey.

### ART provision, regimens, eligibility and uptake

A full description of methods used to estimate ART need and ART uptake in the four DSS are provided elsewhere in this supplement (Wringe *et al.* 2012; Zaba *et al.* 2012), and we include a brief summary of key findings here. In all sites, ART was provided through the public health system and was free at the point of care.

In Karonga, public-sector provision of ART began in the district in June 2005 at a clinic about 200 km from the DSS area, and within the DSS area in October 2006. By 2008, ART uptake was estimated to be approximately 60% of those in need (Wringe *et al.* 2012). The first-line ART regimen was a fixed-dose combination tablet of stavudine, lamivudine and nevirapine, taken twice daily, for the time period covered by our analysis. Individuals were eligible to start ART if they were in WHO clinical stage 3 or 4, or had a CD4 count of <250 cells/mm^3^.

In Kisesa, free ART provision to individuals living in the DSS area started in April 2005. Up to 2008, treatment was provided from a regional referral hospital 20 km away in Mwanza City. Decentralisation of ART provision has proceeded relatively slowly, and ART uptake was estimated as around 3% of those in need in 2007, and very low for both men and women (Wringe *et al.* 2012). The first-line ART regimen was a fixed-dose combination tablet of stavudine, lamivudine and nevirapine during the period up to 2007. Individuals were eligible to start ART if they had a CD4 count of <250 cells/mm^3^ or were in WHO clinical stage 4, and/or had particular WHO clinical stage 3 conditions.

In Masaka DSS, free ART provision within the DSS began in January 2004, provided from a single clinic. By 2008, ART uptake was estimated to be around 70% of those in need (Wringe *et al.* 2012). Individuals were eligible to start ART if they had a CD4 cell count of <200 cells/mm^3^, WHO clinical stage 4 disease or advanced stage 3 disease with persistent or recurrent oral thrush and invasive bacterial infections regardless of CD4 count. First-line treatment consists of a combination of zidovudine, lamivudine and nevirapine.

In the Africa Centre DSS, ART is provided in the public sector through a decentralised network of primary health care clinics (Herbst *et al.* 2009), and by 2009, there were six clinics providing ART in the DSS area. In 2008, 21% of all HIV-infected adults were receiving ART (Cooke *et al.* 2010), while a study based on CD4 counts in the people participating in HIV testing in the surveillance showed that 75% of those needing ART received the treatment in 2010 (Malaza *et al.* 2011). The first-line ART regimen is stavudine and lamivudine, combined with either nevirapine or efavirenz. Individuals were eligible to start ART if they had a CD4 count of <200 cells/mm^3^ and/or were in WHO clinical stage 4.

### Verbal autopsies

In Karonga and the Africa Centre, verbal autopsies have been conducted for all deaths since the establishment of the DSS, and the way in which verbal autopsies are performed is described fully elsewhere (Hosegood *et al.* 2004; Floyd *et al.* 2010). AIDS and non-AIDS deaths can be distinguished by the reviewing clinician, and in the analyses presented here, deaths attributed to TB/AIDS are included as AIDS mortality.

### Statistical analysis

Analysis covered the time period 5 years prior to the start of public-sector ART provision in the study district, and 4–6 years afterwards (Table 1). Analyses were restricted to 15–59 year olds, because in the pre-ART period, a very low proportion of deaths in older individuals were attributable to AIDS.

**Table 1:** Calendar time periods included in analysis of adult mortality

	Kisesa		Masaka		Africa Centre		Karonga	
Pre-ART	Jan 2000	End Dec 2004	Jan 1999	End Dec 2003		End Dec 2003	Aug 2002	End June 2005
ART period 1	Jan 2005	End Dec 2005	Jan 2004	End Dec 2005	Jan 2004	End Dec 2005	July 2005	End Sept 2006
ART period 2	Jan 2006	Jan 2010	Jan 2006	End Dec 2009	Jan 2006	End Dec 2009	Oct 2006*	End Dec 2009

ART, antiretroviral therapy; DSS, demographic surveillance sites.

* A clinic providing ART opened in the DSS area at the end of September 2006.

For each site, person-time was calculated from the date an individual was first resident in the DSS until the earliest of date of death, out-migration from the study area or end of the follow-up period. Person-time was left-truncated if it was more than 5 years prior to the roll-out of free ART. If an individual left and later returned to live in the DSS area, then the time that they were away was not counted towards their person-time, except if they missed only one round of demographic surveillance.

For each site, calendar time was divided into three time periods (Table 1): pre-ART roll-out in the district, early in the time period following ART roll-out (denoted ART period 1) and later in the time period following ART roll-out (denoted ART period 2). Area of residence was categorised into one of ‘remote rural’, ‘roadside rural/peri-urban’ and ‘urban’. For each site, Poisson regression was used to calculate rate ratios (RRs) for the effect of time period, overall and stratified by age, sex and area of residence.

In Kisesa and Masaka, all available data on HIV test results were used to calculate the date of an individual’s first HIV-negative, last HIV-negative and first HIV-positive test result. Person-time before the first HIV test result was not included in analysis, and for individuals who were known to have sero-converted, their sero-conversion date was calculated as halfway between their last HIV-negative and first HIV-positive test result. For individuals with at least one HIV-negative test result, who were not known to have sero-converted, the analysis included their person-time in the HIV-negative population up to 5 years after the last recorded HIV-negative test result.

For Karonga and the Africa Centre DSS, for analysis of AIDS mortality, deaths because of non-AIDS and non-specifiable causes were censored; for analysis of non-AIDS mortality, deaths because of AIDS were censored (Herbst *et al.* 2009; Floyd *et al.* 2010).

## Results

### All-cause mortality among 15–59 year olds, overall

Overall, all-cause mortality fell progressively following ART roll-out in Karonga, Kisesa and Masaka, and by ART period 2 in the Africa Centre DSS (Table 2). This overall trend was seen for both men and women and for all three age groups. In Karonga and Kisesa, the fall in mortality was smaller in remote rural areas, while in the Africa Centre DSS, there was no evidence of a fall in mortality in urban areas.

**Table 2:** All-cause mortality among individuals aged 15–59 years old, by calendar time period

	Kisesa			Masaka			Africa Centre			Karonga		
				
	Deaths	Person years*	Death rate†	Deaths	Person years	Death rate	Deaths	Person years	Death rate	Deaths	Person years	Death rate
ART period												
Pre-ART	528	58.0	9.1	390	35.6	11.0	2484	133.5	18.6	288	28.3	10.2
ART period 1	100	12.7	7.9	71	7.7	9.2	1280	67.4	19.0	158	18.5	8.5
ART period 2	245	34.3	7.2	225	33.7	6.7	2066	139.9	14.8	296	50.2	5.9
Sex												
Male												
Pre-ART	263	28.4	9.3	185	17.2	10.8	1204	57.2	21.0	130	13.5	9.6
ART period 1	51	6.2	8.2	34	3.7	9.3	568	28.7	19.8	84	8.8	9.5
ART period 2	136	16.3	8.3	109	15.9	6.9	998	59.6	16.7	153	23.8	6.4
Female												
Pre-ART	265	29.6	9.0	205	18.5	11.1	1280	76.2	16.8	158	14.8	10.7
ART period 1	49	6.5	7.5	37	4.0	9.2	712	38.7	18.4	74	9.7	7.6
ART period 2	109	17.9	6.1	116	17.7	6.5	1068	80.3	13.3	143	26.5	5.4
Age (years)												
15–29												
Pre-ART	145	31.4	4.6	109	20.5	5.3	730	76.2	9.6	59	16.4	3.6
ART period 1	30	6.8	4.4	21	4.3	4.9	351	39.0	9.0	31	10.6	2.9
ART period 2	59	16.5	3.6	67	18.0	3.7	556	80.7	6.9	64	28.0	2.3
30–44												
Pre-ART	238	18.1	13.2	192	9.9	19.5	1156	37.0	31.2	147	7.9	18.7
ART period 1	45	4.1	11.0	34	2.3	15.0	599	17.5	34.3	79	5.4	14.7
ART period 2	106	11.3	9.4	84	10.1	8.4	848	35.6	23.8	138	15.3	9.1
45–59												
Pre-ART	145	8.4	17.2	89	5.3	16.7	598	20.2	29.6	82	4.0	20.4
ART period 1	25	1.9	13.3	16	1.2	13.5	330	11.0	30.1	48	2.5	19.0
ART period 2	80	6.5	12.3	74	5.6	13.2	662	23.6	28.1	94	7.0	13.4
Area of residence‡												
Remote rural												
Pre-ART	254	30.3	8.4				1399	79.5	17.6	93	13.5	6.9
ART period 1	48	6.5	7.3				672	39.2	17.1	73	9.6	7.6
ART period 2	142	18.9	7.5				1108	77.7	14.3	134	26.3	5.1
Urban												
Pre-ART							105	9.3	11.3			
ART period 1							51	4.2	12.3			
ART period 2							142	12.2	11.6			
Rural, roadside/peri-urban												
Pre-ART	274	27.7	9.9				903	42.7	21.1	195	14.8	13.2
ART period 1	52	6.2	8.4				456	21.0	21.7	85	8.9	9.5
ART period 2	103	15.3	6.7				685	42.7	16.0	162	24.0	6.8

ART, antiretroviral therapy; DSS, demographic surveillance sites.

* Person-years are in units of 1000, for example 58.0 is 58 000 person-years. Table includes all adults aged 15–59 years old in the analysis, irrespective of whether HIV status is known.

† Death rate is per 1000 person-years.

‡ For the Africa Centre, ‘urban’ is defined as areas proclaimed as such by the district municipality; ‘peri-urban’ areas are areas of informal settlement with a population density in excess of 400 persons per km^2^; ‘rural’ areas are the remaining parts of the surveillance area with scattered homesteads and a population density of <400 persons per km^2^. High density settlements (peri-urban) are generally surrounding existing urban areas, or along larger roads in the area. The Masaka DSS is in a very rural area, and so no distinctions were made according to area of residence, while the Karonga and Kisesa DSS have no urban areas.

The overall rate ratio, comparing ART period 2 with the pre-ART period, was 0.58 (5.9 compared with 10.2 deaths per 1000 person-years, 95% CI for the RR [0.50–0.69], *P* < 0.001) in Karonga, 0.79 (7.2 compared with 9.1 deaths per 1000 person-years, 95% CI for the RR [0.67–0.91], *P* = 0.002) in Kisesa, 0.61 (6.7 compared with 11.0 deaths per 1000 person-years, 95% CI for the RR [0.52–0.72], *P* < 0.001) in Masaka and 0.79 (14.8 compared with 18.6 deaths per 1000 person-years, 95% CI for the RR [0.75–0.84], *P* < 0.001) in the Africa Centre DSS. These overall rate ratios changed little when adjusted for age, sex and area of residence (data not shown).

### All-cause mortality among 15–59 year olds, stratified by individual HIV status (Kisesa and Masaka)

In Masaka, the fall in all-cause mortality was dramatic among HIV-positive individuals, with little change among HIV-negative individuals (*P* < 0.001 for interaction, Table 3a). The dramatic falls in mortality among HIV-positive individuals were seen for both men and women, and in each age group. The rate ratio in HIV-positive individuals, comparing ART period 2 with the pre-ART period, was 0.35 (37.8 deaths compared with 108.2 deaths per 1000 person-years). The patterns by HIV status and age were similar when carried out separately for men and women to those seen overall (data not shown).

**Table 3:** All-cause mortality among individuals aged 15–59 years old, by calendar time period and stratified by individual HIV status, Kisesa and Masaka DSS

	HIV-negative					HIV-positive				
		
	Deaths	Person years	Death rate	Crude RR	95% CI	Deaths	Person years	Death rate	Crude RR	95% CI
(a) *Masaka*										
ART period										
Pre-ART	100	26.6	3.8			215	2.0	108.2		
ART period 1	23	6.1	3.8	1.00	0.63–1.57	38	0.5	81.8	0.76	0.54–1.07
ART period 2	91	26.1	3.5	0.93	0.70–1.23	86	2.3	37.8	0.35	0.27–0.45
Sex										
Male										
Pre-ART	55	12.6	4.4	1		98	0.8	116.3	1	
ART period 1	11	2.9	3.8	0.88	0.46–1.67	16	0.2	86.5	0.74	0.44–1.26
ART period 2	47	12.1	3.9	0.89	0.60–1.31	39	0.9	45.6	0.39	0.27–0.57
Female										
Pre-ART	45	13.9	3.2	1		117	1.1	102.2	1	
ART period 1	12	3.2	3.7	1.15	0.61–2.17	22	0.3	78.7	0.77	0.49–1.21
ART period 2	44	13.9	3.2	0.98	0.65–1.48	47	1.4	33.1	0.32	0.23–0.45
Age (years)										
15–29										
Pre-ART	35	15.7	2.2	1		52	0.7	71.4	1	
ART period 1	8	3.5	2.3	1.02	0.47–2.20	10	0.2	63.6	0.89	0.45–1.75
ART period 2	30	14.6	2.1	0.92	0.57–1.50	21	0.6	33.2	0.46	0.28–0.77
30–44										
Pre-ART	33	6.8	4.9	1		121	1.0	121.0	1	
ART period 1	4	1.6	2.4	0.5	0.18–1.41	25	0.3	99.5	0.82	0.53–1.26
ART period 2	23	7.1	3.2	0.66	0.39–1.13	39	1.2	31.5	0.26	0.18–0.37
45–59										
Pre-ART	32	4.1	7.8	1		42	0.3	161.7	1	
ART period 1	11	1.0	11.5	1.46	0.74–2.90	3	0.1	53.7	0.33	0.10–1.07
ART period 2	38	4.4	8.7	1.11	0.70–1.78	26	0.4	64.0	0.4	0.24–0.65
(b) *Kisesa*										
ART Period										
Pre-ART	149	29.2	5.1			128	2.1	62.1		
ART period 1	36	6.4	5.6	1.10	0.76–1.58	22	0.5	48.1	0.77	0.49–1.22
ART period 2	118	23.0	5.1	1.01	0.79–1.28	71	1.7	43.0	0.69	0.52–0.93
Sex										
Male										
Pre-ART	63	13.6	4.6	1		63	0.9	71.4	1	
ART period 1	20	3.0	6.8	1.46	0.88–2.41	10	0.2	51.3	0.72	0.37–1.40
ART period 2	70	10.7	6.6	1.42	1.01–1.99	34	0.6	54.3	0.76	0.50–1.15
Female										
Pre-ART	86	15.6	5.5	1		65	1.2	55.2	1	
ART period 1	16	3.5	4.6	0.84	0.49–1.43	12	0.3	45.7	0.83	0.45–1.53
ART period 2	48	12.4	3.9	0.71	0.50–1.01	37	1.0	36.1	0.66	0.44–0.98
Age (years)										
15–29										
Pre-ART	27	13.9	1.9	1		29	0.8	35.3	1	
ART period 1	10	3.1	3.3	1.68	0.81–3.46	6	0.2	37.0	1.05	0.44–2.52
ART period 2	32	11.1	2.9	1.49	0.90–2.49	12	0.5	26.6	0.75	0.38–1.47
30–44										
Pre-ART	57	10.3	5.6	1		68	1.0	71.2	1	
ART period 1	15	2.2	6.8	1.22	0.69–2.15	9	0.2	39.8	0.56	0.28–1.12
ART period 2	42	7.4	5.7	1.02	0.68–1.51	40	0.9	45.7	0.64	0.43–0.95
45–59										
Pre-ART	65	5.1	12.9	1		31	0.3	108.7	1	
ART period 1	11	1.1	9.6	0.75	0.39–1.41	7	0.1	100.8	0.93	0.41–2.10
ART period 2	44	4.6	9.7	0.75	0.51–1.10	19	0.3	58.7	0.54	0.31–0.96
Area of residence										
Remote rural										
Pre-ART	89	18.4	4.9	1		57	1.0	58.2	1	
ART period 1	15	3.7	4.1	0.84	0.49–1.45	9	0.2	42.0	0.72	0.36–1.46
ART period 2	77	13.4	5.7	1.18	0.87–1.61	32	0.8	41.3	0.71	0.46–1.10
Rural, roadside										
Pre-ART	60	10.9	5.5	1		71	1.1	65.7	1	
ART period 1	21	2.8	7.6	1.38	0.84–2.27	13	0.2	53.5	0.81	0.45–1.47
ART period 2	41	9.6	4.3	0.77	0.52–1.15	39	0.9	44.5	0.68	0.46–1.00

ART, antiretroviral therapy.

In Kisesa, mortality fell in the HIV-positive individuals with no change in mortality in the HIV-negative individuals (*P* < 0.001 for interaction). But the fall in the HIV-positive individuals was not as dramatic as in Masaka, and the difference in trends between HIV-positive and HIV-negative individuals was only seen in men (*P* < 0.001), not in women (*P* = 0.47) (Table 3b). There was weak evidence that mortality among HIV-negative men increased.

### AIDS and non-AIDS mortality among 15–59 year olds (Karonga and Africa Centre)

In Karonga, AIDS mortality fell dramatically following ART roll-out, overall, in men and women, in all age groups and in remote rural and roadside areas (Table 4a). In ART period 2, the rate ratio compared with the pre-ART time period was 0.36 (2.3 compared with 6.4 deaths per 1000 person-years). There was little change over time in non-AIDS mortality (3.6 compared with 3.8 deaths per 1000 person-years in ART period 2 and the pre-ART period, respectively).

**Table 4:** AIDS and non-AIDS mortality among individuals aged 15–59 years old, by calendar time period, Karonga and Africa Centre DSS

	Non-AIDS mortality					AIDS mortality*				
		
	Deaths	Person years	Death rate	Crude RR	95% CI	Deaths	Person years	Death rate	Crude RR	95% CI
*(a) Karonga*										
ART period										
Pre-ART	108	28.32	3.81			180	28.32	6.36		
ART period 1	71	18.52	3.83	1.01	0.75–1.36	87	18.52	4.7	0.74	0.57–0.95
ART period 2	181	50.24	3.61	0.95	0.75–1.20	115	50.24	2.29	0.36	0.29–0.46
Sex										
Male										
Pre-ART	63	13.5	4.7	1		67	13.49	5.0	1	
ART period 1	41	8.8	4.7	1	0.67–1.48	43	8.81	4.9	0.98	0.67–1.44
ART period 2	92	23.8	3.9	0.83	0.60–1.14	61	23.76	2.6	0.52	0.37–0.73
Female										
Pre-ART	45	14.8	3.0	1		113	14.83	7.6	1	
ART period 1	30	9.7	3.1	1.02	0.64–1.62	44	9.71	4.5	0.59	0.42–0.84
ART period 2	89	26.5	3.4	1.11	0.77–1.59	54	26.48	2.0	0.27	0.19–0.37
Age (years)										
15–29										
Pre-ART	26	16.4	1.6	1		33	16.42	2.0	1	
ART period 1	17	10.6	1.6	1.01	0.55–1.86	14	10.61	1.3	0.66	0.35–1.23
ART period 2	53	28.0	1.9	1.2	0.75–1.91	11	27.96	0.4	0.2	0.10–0.39
30–44										
Pre-ART	45	7.9	5.7	1		102	7.88	12.9	1	
ART period 1	31	5.4	5.8	1.01	0.64–1.59	48	5.38	8.9	0.69	0.49–0.97
ART period 2	70	15.3	4.6	0.8	0.55–1.17	68	15.25	4.5	0.34	0.25–0.47
45–59										
Pre-ART	37	4.0	9.2	1		45	4.02	11.2	1	
ART period 1	23	2.5	9.1	0.99	0.59–1.66	25	2.53	9.9	0.88	0.54–1.44
ART period 2	58	7.0	8.3	0.9	0.59–1.35	36	7.03	5.1	0.46	0.30–0.71
Area of residence										
Remote rural										
Pre-ART	36	13.49	2.67	1		57	13.49	4.22	1	
ART period 1	39	9.6	4.06	1.52	0.97–2.40	34	9.6	3.54	0.84	0.55–1.28
ART period 2	82	26.25	3.12	1.17	0.79–1.73	52	26.25	1.98	0.47	0.32–0.68
Rural, roadside										
Pre-ART	72	14.83	4.86	1		123	14.83	8.29	1	
ART period 1	32	8.92	3.59	0.74	0.49–1.12	53	8.92	5.94	0.72	0.52–0.99
ART period 2	99	23.99	4.13	0.85	0.63–1.15	63	23.99	2.63	0.32	0.23–0.43
*(b) Africa Centre*										
ART period										
Pre-ART	670	133.5	5.0	1		1814	133.45	13.6	1	
ART period 1	379	67.4	5.6	1.12	0.99–1.27	901	67.39	13.4	0.98	0.91–1.07
ART period 2	679	139.9	4.9	0.97	0.87–1.08	1387	139.91	9.9	0.73	0.68–0.78
Sex										
Male										
Pre-ART	402	57.2	7.0	1		802	57.24	14.0	1	
ART period 1	203	28.7	7.1	1.01	0.85–1.19	365	28.69	12.7	0.91	0.80–1.03
ART period 2	376	59.6	6.3	0.90	0.78–1.03	622	59.6	10.4	0.74	0.67–0.83
Female										
Pre-ART	268	76.2	3.5	1		1012	76.21	13.3	1	
ART period 1	176	38.7	4.6	1.29	1.07–1.56	536	38.71	13.9	1.04	0.94–1.16
ART period 2	303	80.3	3.8	1.07	0.91–1.26	765	80.3	9.5	0.72	0.65–0.79
Age (years)										
15–29										
Pre-ART	184	76.2	2.4	1		546	76.22	7.2	1	
ART period 1	121	39.0	3.1	1.29	1.02–1.62	230	38.97	5.9	0.82	0.71–0.96
ART period 2	214	80.7	2.7	1.10	0.90–1.34	342	80.7	4.2	0.59	0.52–0.68
30–44										
Pre-ART	242	37.0	6.5	1		914	37.01	24.7	1	
ART period 1	132	17.5	7.6	1.16	0.93–1.43	467	17.47	26.7	1.08	0.97–1.21
ART period 2	195	35.6	5.5	0.84	0.69–1.01	653	35.62	18.3	0.74	0.67–0.82
45–59										
Pre-ART	244	20.2	12.1	1		354	20.22	17.5	1	
ART period 1	126	11.0	11.5	0.95	0.77–1.18	204	10.96	18.6	1.06	0.90–1.26
ART period 2	270	23.6	11.5	0.95	0.80–1.13	392	23.59	16.6	0.95	0.82–1.10
Area of residence										
Remote rural										
Pre-ART	376	79.5	4.7	1		1023	79.45	12.9	1	
ART period 1	199	39.2	5.1	1.07	0.90–1.27	473	39.23	12.1	0.94	0.84–1.04
ART period 2	378	77.7	4.9	1.03	0.89–1.19	730	77.67	9.4	0.73	0.66–0.80
Urban										
Pre-ART	38	9.3	4.1	1		67	9.27	7.2	1	
ART period 1	16	4.2	3.9	0.94	0.52–1.68	35	4.15	8.4	1.17	0.77–1.75
ART period 2	48	12.2	3.9	0.96	0.63–1.47	94	12.21	7.7	1.06	0.78–1.46
Peri-Urban										
Pre-ART	244	42.7	5.7	1		659	42.72	15.4	1	
ART period 1	135	21.0	6.4	1.13	0.91–1.39	321	21.01	15.3	0.99	0.87–1.13
ART period 2	208	42.7	4.9	0.85	0.71–1.03	477	42.71	11.2	0.72	0.64–0.81

ART, antiretroviral therapy; DSS, demographic surveillance sites.

* AIDS mortality rates are much lower than mortality rates in HIV-positive individuals, because the denominator is the total population and not just those who are HIV-positive. This also explains the much higher mortality rates in the Africa Centre DSS than in the Karonga DSS, because HIV prevalence is higher in the Africa Centre DSS than in the Karonga DSS.

In the Africa Centre DSS, AIDS mortality fell overall, for men and women, among 15–44 year olds but not among older individuals, and in rural and peri-urban but not urban areas (Table 4b). Overall, the rate ratio comparing ART period 2 with the pre-ART period was 0.73 (9.9 compared with 13.6 deaths per 1000 person-years). Non-AIDS mortality fluctuated, but there was little evidence of a trend over time (Table 4b).

## Discussion

There is strong evidence of a fall in all-cause mortality among 15–59 year olds after ART roll-out in all four DSS included in this study. In Karonga and Masaka DSS, the fall was around 40%, and in Kisesa and the Africa Centre DSS around 20%, during the period 2–5 years following ART roll-out. For three sites, supporting evidence that this fall is largely attributable to ART provision comes from analysis of AIDS and non-AIDS mortality (Karonga and the Africa Centre), or from mortality trends stratified by individual HIV status (Masaka). In contrast, in Kisesa, the fall in all-cause mortality was similar for HIV-positive and HIV-negative women, while the evidence for different mortality trends in HIV-positive and HIV-negative men was due partly to increased mortality among HIV-negative men for which there is no obvious explanation.

Our findings build on those already published from the same DSS (Jahn *et al.* 2008; Herbst *et al.* 2009; Floyd *et al.* 2010), and are consistent with others that indicate a large effect of ART provision on all-cause and AIDS mortality (Reniers *et al.* 2009; Mwagomba *et al.* 2010). An important strength of our new analysis is that it updates earlier work and standardises methods and presentation of findings across four DSS to enable fair comparison and synthesis of findings.

The overall findings fit reasonably well with what is known about ART uptake and retention in treatment programmes in each site, and with what was known about the population-attributable fraction (PAF) of all-cause mortality because of AIDS prior to ART provision. By multiplying together the pre-ART fraction of deaths attributable to AIDS (*f*), the ART uptake as a proportion of those in need of treatment (*u*) and retention in care – the proportion alive and on treatment after 1 year (*r*), we can approximate the proportion of deaths averted by the use of ART (*p*). Symbolically, *p*≍*f* × *u* × *r*.

In Karonga, 63% of deaths among 15–59 year olds were attributed to AIDS prior to ART provision (Jahn *et al.* 2008); ART uptake as a proportion of those in need has been estimated to be at least 60% by 2008 (Wringe *et al.* 2012), and among patients who first registered at the ART clinic in the study area after January 2008, retention in care at 12 months after starting ART was 88% (C. Mwafulirwa, unpublished data), giving an approximate estimate of the proportion of deaths averted by ART of 33% (=0.63 × 0.60 × 0.88), compared with an observed fall of 42%.

For Masaka, the proportion of deaths attributable to AIDS in the pre-ART era was 46% (Porter & Zaba 2004); ART uptake was estimated to be at least 70% of those in need by 2008 (Wringe *et al.* 2012); and retention in care is 87% in Masaka at 12 months after starting ART (P. Kazooba, unpublished data), so an approximate estimate of the proportion of deaths averted by ART is 28% (=0.46 × 0.7 × 0.87), again somewhat lower than the observed fall (39%).

In the Africa Centre DSS, 60% of deaths among 15–59 year olds were attributed to AIDS prior to ART provision (Hosegood *et al.* 2004) – lower than in Karonga despite much higher HIV prevalence, probably because the mortality rate for non-HIV-related causes of death is higher. The percentage of HIV-positive individuals in need of ART was 45% (Zaba *et al.* 2012), while 21% of HIV-positive individuals were on ART in 2008 (Cooke *et al.* 2010), so that uptake as a proportion of need was 47% (=21/45). Assuming retention in care is 75%, then a very approximate estimate of the proportion of deaths averted by ART would be 21% (=0.60 × 0.47 × 0.75), similar to the observed fall.

In contrast, in Kisesa, ART uptake was 3% of those in need at the end of 2007, so ART provision cannot have had much effect on population-level mortality.

In conclusion, our study provides strong evidence that public-sector ART provision has brought substantial reductions in adult mortality. The largest falls have occurred where ART provision has been rapidly decentralised (Karonga and the Africa Centre DSS) or where it has been available locally (Masaka), suggesting that decentralisation and/or localisation of treatment provision are important. There remains scope to increase uptake of ART among individuals in need of treatment, and thus bring even larger benefits in terms of lives saved.
